# Unusual Driver Behavior Detection in Videos Using Deep Learning Models

**DOI:** 10.3390/s23010311

**Published:** 2022-12-28

**Authors:** Hamad Ali Abosaq, Muhammad Ramzan, Faisal Althobiani, Adnan Abid, Khalid Mahmood Aamir, Hesham Abdushkour, Muhammad Irfan, Mohammad E. Gommosani, Saleh Mohammed Ghonaim, V. R. Shamji, Saifur Rahman

**Affiliations:** 1 Computer Science Department, College of Computer Science and Information Systems, Najran University, Najran 61441, Saudi Arabia; 2 Department of Computer Science and Information Technology, University of Sargodha, Sargodha 40100, Pakistan; 3 Department of Computer Science, University of Management & Technology, Lahore 54770, Pakistan; 4 Nautical Science Department, Faculty of Maritime Studies, King Abdulaziz University, Jeddah 22254, Saudi Arabia; 5 Faculty of Computer Science and Information Technology, Virtual University of Pakistan, Lahore 54000, Pakistan; 6 Electrical Engineering Department, College of Engineering, Najran University, Najran 61441, Saudi Arabia; 7 Department of Hydrographic Surveying, Faculty of Maritime Studies, King Abdulaziz University, P.O. Box 80401, Jeddah 21589, Saudi Arabia

**Keywords:** abnormal behaviors, drowsiness, driver, deep learning, human activity, surveillance

## Abstract

Anomalous driving behavior detection is becoming more popular since it is vital in ensuring the safety of drivers and passengers in vehicles. Road accidents happen for various reasons, including health, mental stress, and fatigue. It is critical to monitor abnormal driving behaviors in real time to improve driving safety, raise driver awareness of their driving patterns, and minimize future road accidents. Many symptoms appear to show this condition in the driver, such as facial expressions or abnormal actions. The abnormal activity was among the most common causes of road accidents, accounting for nearly 20% of all accidents, according to international data on accident causes. To avoid serious consequences, abnormal driving behaviors must be identified and avoided. As it is difficult to monitor anyone continuously, automated detection of this condition is more effective and quicker. To increase drivers’ recognition of their driving behaviors and prevent potential accidents, a precise monitoring approach that detects abnormal driving behaviors and identifies abnormal driving behaviors is required. The most common activities performed by the driver while driving is drinking, eating, smoking, and calling. These types of driver activities are considered in this work, along with normal driving. This study proposed deep learning-based detection models for recognizing abnormal driver actions. This system is trained and tested using a newly created dataset, including five classes. The main classes include Driver-smoking, Driver-eating, Driver-drinking, Driver-calling, and Driver-normal. For the analysis of results, pre-trained and fine-tuned CNN models are considered. The proposed CNN-based model and pre-trained models ResNet101, VGG-16, VGG-19, and Inception-v3 are used. The results are compared by using the performance measures. The results are obtained 89%, 93%, 93%, 94% for pre-trained models and 95% by using the proposed CNN-based model. Our analysis and results revealed that our proposed CNN base model performed well and could effectively classify the driver’s abnormal behavior.

## 1. Introduction

Deep Learning is critical in Intelligent Transportation systems for vehicle identification, traffic forecast, visual task recognition, incident traffic interference, traffic signal timing, and recognition of abnormal driving behaviors. Currently, recognizing human activity in surveillance videos is a popular research area. Computer vision and machine learning techniques identify and recognize human activities. Recognizing social movements also entails categorizing them as usual, suspicious or abnormal. Human behaviors and actions can be understood using a variety of video qualities that help classify activities as normal or abnormal. While certain activities are exceptional in one circumstance, they may be considered regular in another. Given the multiple hurdles that must be overcome when recognizing action and human behaviors, the importance of recognizing human activity has grown. Abnormal activities are referred to as unusual or suspicious.

Situations such as an abandoned object, a patient falling in the hospital, students cheating during exams [1], driver drowsiness [2], road accidents, traffic rules violations, and in public places such as slapping, punching, hitting, firing, abuse, snatching, fighting, terrorism, robbery, illegal parking, fire disaster. According to World Health Organization (WHO) statistics, road accidents are currently one of the top ten reasons for death worldwide [3]. According to studies, human activities such as drivers’ irregular driving behaviors cause most road accidents [4]. Abnormal driving is defined by the International Organization for Standardization (ISO) as a situation in which a driver’s capability to drive is reduced due to their attention being diverted from regular driving. As a result, irregular driving behaviors in drivers must be detected and either alerted or reported to the Transportation Regional office for recording. The increased usage of vehicles has negative consequences such as traffic congestion, accidents, injuries, deaths, and economic loss. Driver behavior is one of the most critical aspects of determining road safety. As a result, driver behaviors monitoring and detection systems have recently gained popularity as research topics. It displays several symptoms representing the driver’s state, such as facial expressions, eye-opening, and closing. To raise drivers’ awareness of their driving patterns and prevent future automotive accidents, we must investigate a fine-grained monitoring system that detects and recognizes aberrant driving behaviors, such as eating, drinking, smoking, or sleeping. 

There are three kinds of abnormal driving behaviors. The first is a distracting driving activity that satisfies the driver’s physical needs, such as eating, drinking, smoking, or adjusting the air conditioning. The second goal is to satisfy the driver’s desire to engage in distracting driving behaviors like talking on the phone or using unnecessary equipment. The third category includes distracted driving behaviors influenced by the environment, such as caring for children or paying long-term attention to dramatic situations outside the automobile. Among the above abnormal driving behaviors, mobile phones have emerged as an essential component in modern abnormal driving [5].

In a current simulation, researchers discovered that talking on the phone [6] while driving can affect a driver to lose 20% of their concentration. If the content of the conversation is critical, it can cause up to 37% distraction, making the driver 23 times more expected to have an accident than average drivers. As a result, using a cell phone while driving is considered a critical problematic driving behavior for automated detection in this study [7]. 

Improper driving [8] practices increase the chances of an accident occurring. One of the critical areas of focus is thus detecting driving behaviors. Driving monitoring systems alert the driver to possibly risky driving by identifying and distinguishing between regular and unsafe driving. The motorist can improve their driving technique and reduce their risk of accident involvement. A driving monitoring system’s precision is a vital component. Various approaches have been used to identify driver behaviors depending on the system’s goals. Sensors are significantly used in driving monitoring systems. It is vital to be aware of acts that may cause accidents while driving and to take steps to avoid them. Eye-blinking and moving one’s eyes away from the road are signs of weariness or anxious emotions that should be observed. This is because such activities cause drivers to lose focus on the road, increasing the risk of an accident. There are two types of factors that influence driving behaviors. The first category includes variables that influence driving behaviors, such as physiological characteristics (e.g., circadian rhythm, age, gender) and environmental reasons (i.e., weather and traffic conditions.). The second class includes vehicle-related parameters (such as speed, acceleration, and throttle position) and driver-related information (such as eye movement and blood-alcohol content) derived from specific behaviors.

In most cases, abnormal driving is identified for the second group. Detecting anomalous driving mostly relies on data acquired by several sensors, such as an accelerator angle sensor, brake pedal pressure sensor, and vehicle-borne radar, in real-time and over time. Given the widespread deployment of conventional driving-associated sensors and newly developing signal processing technologies, unlabeled driving-related data is already pervasive, and the transport industry has arrived in the era of big data [9]. The majority of unusual driving behavior is taken into consideration as one of our main strengths. Limitations are based on the visible portion of the driver’s behavior. The main benefits of this research are that the most frequent distraction-causing behaviors were taken into account when creating the dataset. It increases the driver’s concentration while driving and aids in avoiding on-road collisions. The main research contributions are:

- The dataset was developed for driver abnormal activity detection systems. The main unexpected activities of the drivers are included in the datasets. Expert annotators did data labeling for the data set processing.
- We propose using a motion based Keyframe extraction technique to extract only the most critical frames from a video series. The unwanted frames are removed from the processing.
- Results Analysis of Pre-Trained and Fine-Tuned CNN models. Using transfer learning concepts, comparing the performance of pre-trained models to a CNN model.
- Evaluation of the research work using basic evaluation criteria.

The remaining part of the paper is arranged like this: The literature review is discussed in Section 2 on detecting abnormal driver behaviors. Section 3 discusses the proposed methodology. The dataset, experimental setup, and performance evaluation measure are all discussed in Section 4. The empirical findings and discussion are presented in Part 5. The final portion contains the conclusion and future work.

## 2. Literature Review

This section examines existing works on detecting abnormal driving behaviors. Several algorithms for abnormal driving detection from video have been introduced recently. The literature has been discussed based on the driver’s abnormal behaviors, including drowsiness, distracted driver activities, and other abnormal behaviors. 

In [10], Weavers, side slipping, quick U-turns, swerving, turning in such a wide circle, and unexpected braking were all cases of abnormal driving behavior discussed. They proposed a fine-grained identification system that employs various sensors to achieve real-time high-accuracy abnormal driving behavior. First, extract unique features from smartphone gyroscopes and guidance sensor readings to recognize sixteen relevant features for capturing driving behavior patterns. The features are then trained using the SVM machine learning approach to generate a classifier model for fine-grained recognition.

They have proposed [11] Temporal Convolutional Network (TCN) and Soft Thresholding based algorithm for driving behavior to improve the model’s stability and accuracy. Four public data sets have been used to evaluate the proposed model thoroughly. Their experimental results showed that the proposed model outperforms the top baselines available by 2.24%. This work [12] proposed a deep-learning architecture, “DriverRep” for extracting the latent representations associated with each individual. The suggested stacked encoder architecture is then used in a fully unsupervised triplet loss to identify unsupervised triplet samples from data and extract embeddings. To create residual encoder blocks, dilated causal convolutions have been used. The classification accuracy of SVM evaluation findings showed an average accuracy of 94.7% for two-way recognition and 96% for three-way recognition using two datasets containing ten drivers.

Hou, M. et al. [13] proposed a lightweight abnormal driving behavior detection framework, which utilizes intelligence to the edge and provides IoT devices to recognize and process the data. The framework comprises four parts: (a) video retrieval, (b) mask detection, (c) driver anomalous motion detection, and (d) drowsiness driving detection. The video restoration module filters the noisy video, and the mask detection module provides a model based on facial anchor detection. To confirm the accuracy of the dual-stream model, the third module was used to present an algorithm for the bus driver to identify abnormal movements. The fourth module determines whether the driver exhibits any fatigue driving behaviors based on the various elements of the facial Point.

This paper [14] explained the context-awareness perspective by presenting a general architecture of the smart car. A hierarchical context model is suggested to describe the complex driving environment. A software platform is created to offer the context model and applications’ operating environment. Evaluation of performance demonstrates the viability and effectiveness of the suggested strategy for smart cars. This technology, however, is limited to informing the driver and managing the car and does not convey warning signals to other vehicles on the road. Rakotonirainy, A. et al. [15] presented a real-time context-aware method for collecting and analyzing appropriate information about the surroundings, vehicle, and driver. It also collects data from driver surveys to generate driving scenarios. The Bayesian network is utilized to reason about this ambiguous contextual knowledge by observing and predicting the driver’s future behavior through a learning process. Although the technology can predict the driver’s future behavior, it cannot identify the driver’s current state and advise other vehicles on the road.

Sandberg, D. et al. [16] proposed a real-time method for detecting fatigue from collected driver behavior data such as vehicle speed, adjacent position, yawing position, lane position steering, and wheel angle. Their technology uses a combination of tiredness indicators to predict whether a driver is drowsy and, if necessary, send an alert [17]. A non-contact method to avoid driver sleepiness was developed by sensing the driver’s eyes and determining whether they were closed or open with a CCD camera. The device records the driver’s face and uses image processing algorithms to determine if the driver’s eyes are closed for long periods. If the driver’s eyes are closed, it indicates sleepiness, and the system will notify them. Ramzan et al. [18] proposed a framework where the scenario was simulated using the NS3 WSN network simulator. This simulation shows that the accident ratio can be significantly decreased. When a driver’s fatigue is detected, a signal alert is sent to other drivers in nearby vehicles; different sensor nodes are used.

A study by Jeong, M et al. [19] suggested a face landmark detection technique be used in actual driving conditions. The author first uses the nose region as a reference point to determine the distance between a landmark and a reference point. The local weighted random was then used to maintain the generality. They then employ global face models based on the spatial relationship between landmarks to pinpoint the incorrect local landmark positions and reorganize the overall landmark arrangement. According to the literature on automated anomalous driving behavior detection, three detection systems are commonly used. The first is based on using various sensors to detect human physiological signs (such as electrooculograms, electro-encephalograms, blood flow changes, and respiratory. The second is based on facial characteristics (i.e., variations in eye movement, head movement, mouth movement, and hand elements). The third is based on steering wheel motion characteristics, which can be used to determine the driver’s hand pressure, steering time, and braking behavior [20].

Traditionally, driving data are collected primarily from road monitoring equipment or automobile sensors. However, road monitoring technology can only detect abnormal driving behavior in specific locations, and data from multiple sensors cannot be collected consistently, making post-processing difficult [21]. Several studies collect data using cell phones and hand-annotated data to address these concerns to detect abnormal driving behavior [22]. By diverting the accelerometer, Promwongsa et al. [23] presented a method for more accurately calibrating smartphone orientation. Detecting inappropriate driving behavior is frequently regarded as the first challenge in achieving the desired independent driving goal. Safety concerns are undeniably top priorities for the autonomous driving assignment. It is widely acknowledged that driver behavior must be strictly regulated to avoid potential accidents. As a result, many high-resolution cameras installed within the driver’s vehicle may be used to monitor the driver’s condition continuously. In general, images captured by high-resolution cameras must be analyzed in real-time to determine whether the driver’s current condition is normal or not. According to the descriptions above, both the accuracy and the efficiency of anomalous driving behavior detection are highly desired. Furthermore, high-speed wireless transmissions are required to achieve the rapid and dependable transmission of high-level quality videos, supporting the previously mentioned automated aberrant driving behavior identification duty [24].

In [25] GPS, a camera, an alcohol detector, and an accelerometer sensor are utilized to determine whether a driver is alcoholic, tired, or irresponsible. However, all the solutions depend on pre-installed infrastructures and further gear, which come at a cost. Furthermore, additional hardware may be subjected to time differences between day and night, inclement weather, and expensive maintenance costs. 

Drivers’ driving styles are classified as Safe or Unsafe using accelerometers, gyroscopes, and magnetometers. Accelerometers could also detect drunk driving and sudden driving maneuvers.

Peng Ping et al. [26] show that distracted driving is one of the leading causes of road accidents caused by driver carelessness. Distracting activities include safe driving, texting right, drinking, talking to the passenger, left phone usage, hair or makeup, texting left, Reaching behind, and adjusting the radio. The performance measure accuracy has been for each class. 

All activities that distract the driver from normal driving are considered abnormal driving activities. The existing literature focuses on a small number of distraction activities. The most common activities of the driver that distract from normal driving, such as calling, eating, drinking, smoking, and normal with different positions and poses, were not considered altogether in the same study.

## 3. Proposed Methodology

**Figure 1:**
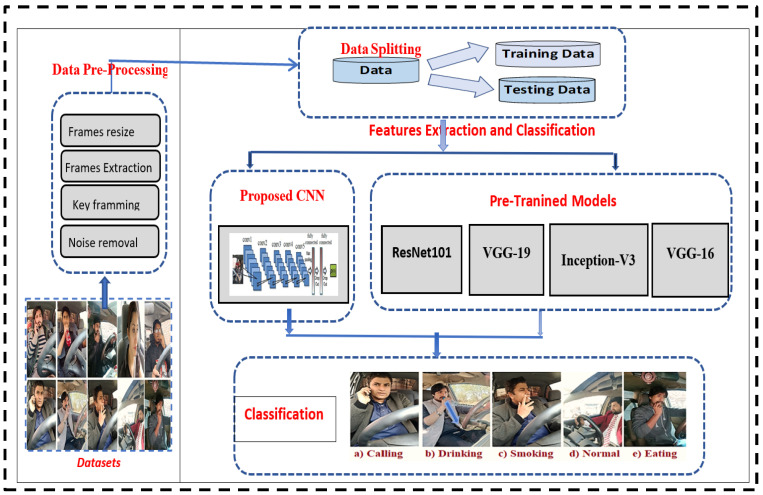
Proposed Framework for Driver Unusual behavior Detection.

Figure 1 demonstrates the proposed system’s framework as well as the stages of the proposed research methodology. Deep learning is used in the proposed method to classify keyframes of a video series in normal and abnormal behaviors. The basic steps are discussed in the next section. 

## 4. Materials and Methods

All of the steps are thoroughly discussed in this section. Dataset collection is the first step, the second step is the pre-processing, followed by key frame extraction, then we utilized deep learning-based models for feature extraction and classification. Python with TensorFlow and Keras was used to implement the framework. The proposed method based on CNN and pre-trained models is implemented using Python 3.7 and performed on a Desktop system Intel core i5-4200 M with 16 GB RAM and GeForce GTX 1080 CPU. 

Pre-trained and proposed CNN-based models with the best configuration are utilized to analyze the results by taking new datasets. The newly created dataset collects the different videos we detect based on the five classes. Furthermore, the image’s noise is removed. Pre-trained models ResNet101, VGG16, VGG19, Inception-v3 and proposed CNN-based models are utilized to analyze this type of dataset. 

### 4.1. Datasets

The existing dataset where the abnormal behaviors of the driver founded are “State Farm Distracted Driver Detection”. The driver’s abnormal behaviors in this dataset which distracted the driver from normal driving, include talking with passengers and phone, texting, normal, drinking, radio sets, hair setting, and makeup. 

A few essential abnormal behaviors of drivers like smoking [27], also needed to be considered for detection. 

This dataset was created and used which includes the most significant abnormal driving activities discovered while driving. The five most common classes driver-smoking, driver-calling, driver-eating, driver-drinking, and driver-normal included in this dataset. For the normal Driver class, the YawDD dataset is also included [28]. Video is acquired using a DSLR camera with a resolution of 20.1 megapixels, an average of 29 frames per second, and a frame size of 1440 × 1080. Video is sliced according to categories, and every clip’s duration is an average of 3 s. For this dataset, the inter reliability of daters for data annotation is assessed using Kappa Statistics. Two raters annotated it, and their inter-reliability using kappa statistics was 95%. Then, samples where there were differences of opinion were discussed, and then the mutual coordination of the rater annotated all the ratings.

The key difficulties with the recognition model include fluctuations in illumination, pose, variations in view angle, camera position, occluded images, and hands or arm positions, which negatively impact the approaches’ performance. These are common challenges of all the classes of the dataset. Changing the viewpoint can significantly impact the perception, hand orientation, and level of occlusion. Occlusion of mobile, cigarettes, eating objects or bottles, and self-occlusion are challenges in correctly detecting unusual activity. Meanwhile, taking, eating, calling, and drinking, the lighting conditions must be maintained. Some challenges are specific to the class, like hand and arm position for eating, calling, drinking, and smoking. These types of challenges for driver abnormal detection are considered and handled in the proposed method. The datasets contain a total number of videos 36. The details of the dataset are shown in Table 1. 

**Figure 2:**
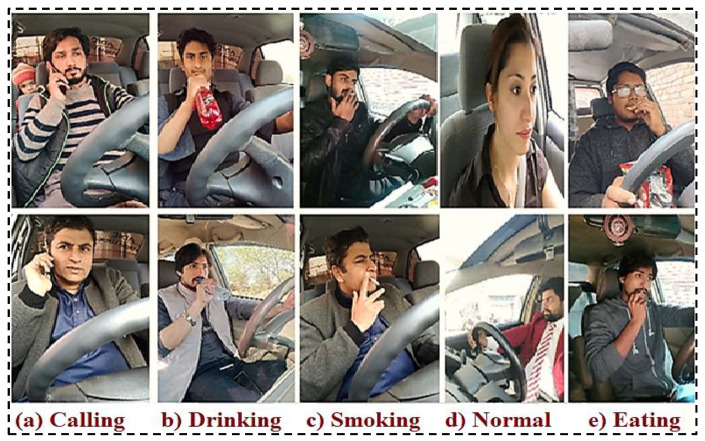
Keyframes for Unusual Activities of Driver.

**Figure 3:**
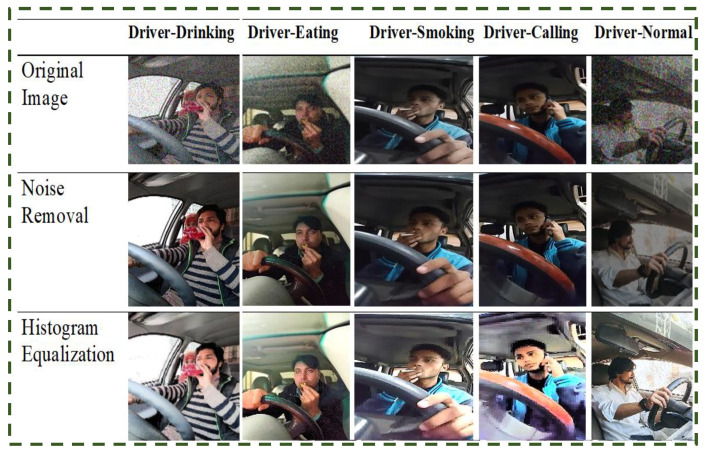
Some raw images with pre-processed images.

Sample images of unusual driver activities processed keyframes are shown in Figure 2 and raw images are shown in Figure 3. 

### 4.2. Video Preprocessing

After being captured, long-length videos are cut into three-second-long clips. Depending on the category of unusual activities, we transform each video into. mp4 format. The Gaussian filter is used for denoising in video preprocessing, and histogram equalization is applied to video frames. 

The quality of the images exhibits significant variety, as can be seen. Images in the dataset come in different sizes, shapes, and resolutions. Additionally, some of the raw images are of quite poor quality. Effective pre-processing methods must be used in order to perform the classification of images. 

As a result, all of the images were scaled to 224 × 224. We tried a variety of image improvement approaches, including the Gaussian Filter and Histogram Equalization, to increase the quality of the images. The histogram equalization is performed on the input drive image to eliminate light intensity variations and increase the overall brightness and contrast of the image. Images from the dataset that were of variable quality were subjected to each of these procedures. Figure 3 displays the outcomes of various methods. After being captured, long-duration videos are converted into frames. The Gaussian filter is used during preprocessing to remove noise from video frames, and histogram equalization is applied to adjust the contrast of the video frames. 

### 4.3. Keyframe Extraction 

All frames extracted in the framing step are used for feature extraction and training in previous techniques. Many consecutive frames are the same. The model’s complexity and computational power increase as the same frames are repeated. The key frame extraction technique will be used in this research work to remove the same consecutive frames from a total video frame. The number of training frames and computational work required to process the same frames is reduced when using key frame extraction [29] (Algorithm 1).

First, to extract the keyframes, we sampled all of the videos to 10 frames using a skipping factor of SF = 5 on four consecutive frames. The skipping factor aids in the elimination of redundant frames. We proposed an approach to extract keyframes based on the motion; in this technique, we take the pixel-wise absolute differences between two successive frames [30].

(1) $${\left(absdiff\right)}_{f}=absdiff\left(Cf_{i+1}\;,\;Pf_{i}\right)$$

where $Pf_{i}$ represents the previous frame and $Cf_{i+1}$ as a current frame in the preceding equation. The average difference is then computed ($Avg_{diff}$) of (*absdiff*)*_f_* matrix.

(2) $$Avg_{diff}=Avg_{f}\left({\left(absdiff\right)}_{f}\right)$$

If the $Avg_{diff}$ going beyond a pre-defined Threshold (*T*), the current frame is chosen as the key or omitted otherwise.

(3) $$KF_{i}=if\left\{\begin{array}{c} Avg_{diff}>T\;key\;frame\;\;\;\;\;\;\;\;\;\;\;\\ Avg_{diff}<T\;Not\;a\;key\;frame \end{array}\right.$$

**Figure 4:**
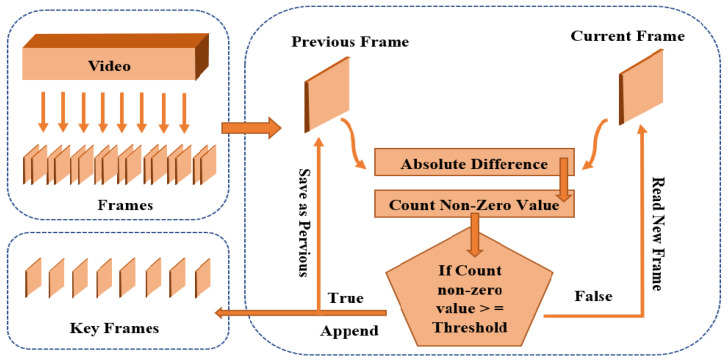
Flow Diagram of Key Frame Extraction Technique.

We revised the frames as prev_frame = curr_frame and repeat the complete procedure. The flow diagram for the keyframes extraction is shown in Figure 4.

**Algorithm 1:** Key-Frame Extraction [29]  Read videos from the directory  Store all videos in an array name all_videos []  Specify a threshold *T* for keyframe selection  for i uptoall_videos in array  Read frame i as prev_frame also do (frameCount) in video (i)  Loop untilwhile (fc < frameCount)
7.  Read the next frame as curr_frame (i + 1)8.  Compute absdiff (curr_frame, prev_frame)9.  Compute the Average of absdiff10.If Averagediff > *T* (threshold)11.Select the current frame as a keyframe12.Update frames: prev_frame = curr_frame13.Update:


The pseudocode for keyframe extraction is discussed above. The algorithm receives all of the video data’s extracted frames and returns a list of keyframes. The first frame of the video is considered a keyframe and is put on the list of keyframes. The frame after that is compared to the frame before that, and the resemblance of successive frames is calculated. This resemblance is based on the absolute difference between two frames, calculated as a non-zero value with a simple matrix subtraction method. To compare non-zero values, a threshold value is used. The threshold is divided into two regions: above and below the threshold (also known as the binary decision threshold). Values less than the threshold indicate the same frames, while those greater than the threshold indicate different frames.

### 4.4. Features Selection and Classification

Deep learning methods combine feature extraction and classification into a single module. This research used the proposed CNN-based and pre-trained deep-learning models for feature selection and classification.

#### 4.4.1. Proposed CNN-Based Architecture

The approach used to carry out the learning process is referred to as the training strategy. The neural network is trained to achieve the least amount of loss. This is performed by finding parameters that suit the neural network for the data collection. The loss index indicates the quality of the representation to be learned and describes the task that the neural network must fulfil. The optimization method changes the parameters of the neural network. There are various optimization algorithms, each with its own set of computational and storage needs. Gradient descent is the most basic fundamental optimization algorithm. The parameters are adjusted in the direction of the gradient of the loss index at each epoch. The optimization approach manages the learning process of a neural network. Gradient descent is the most basic training algorithm. There are numerous optimization algorithms. Memory, processing speed, and numerical accuracy all have different needs.

The proposed CNN-based architecture consists of four convolutional layers to increase the image’s detail. For a given image input, the convolutional layer in the proposed architecture learns 32, 64, 128 filters in parallel. As a result, the proposed models extract characteristics in 32, 64, and 128 in various ways from the input data. In this architecture, the convolutional layers were increased one by one until the desired results were obtained. After the convolutional max pooling process, it uses the rectified layer unit as an activation function in these layers, recognizing the network’s spatial variance. To avoid overfitting, an abstract form of representation is represented using max-pooling.

Additionally, reducing the number of parameters lowers the computational cost. For all max-pooling functions across the network, the stride size is the pool size of 2 × 2. After the third convolutional layer, the pooling function adds a flattening function to convert the frame pixel into a vector column.

**Figure 5:**
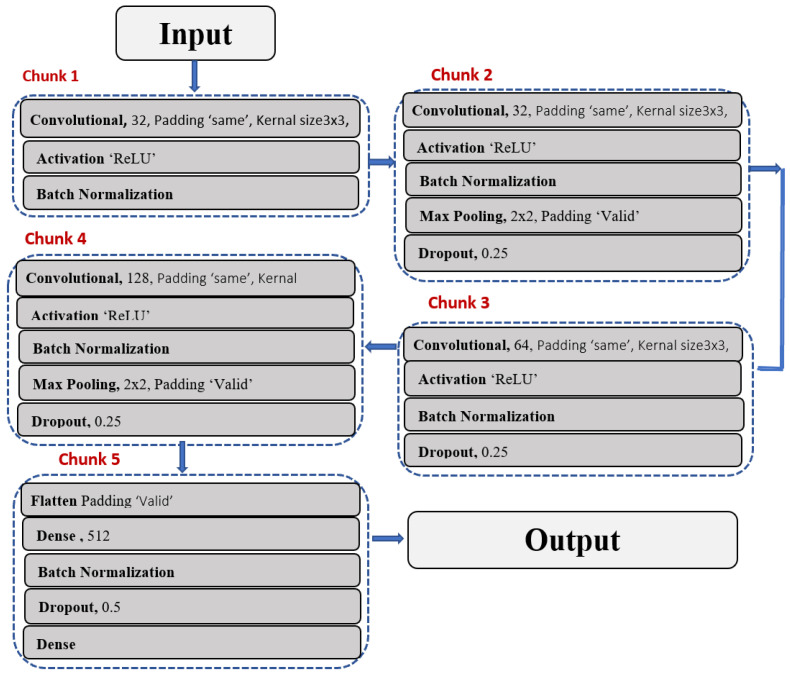
Proposed CNN-based Architecture with organizations of layers of.

This paper suggests a deep learning-based 22-layer CNN architecture for detecting driver abnormal behavior (L1, L2, L3, ……, L22) as shown in Figure 5. The architecture is divided into five sections. The first chunks are composed of three layers: convolutional, activation, and batch normalization (L1, L2, L3). After the input layer performs convolution operations on the input image, the repeating layers in the first four chunks are nearly identical to L1. L2 refers to the ReLu activation function, which breaks network linearity. L3 represents the batch normalization layer, L7 represents the maximum pooling layer, and L8 represents the dropout layer, which is used to remove some instances from the architecture temporarily. Dropping out also keeps the model from becoming overly fit.

#### 4.4.2. Transfer Learning Approach

The Transfer Learning approach is popular in computer vision, and this research focuses on assessing how well pre-trained models perform on image data. The newly created dataset collects the different videos based on the five classes. Also, the image’s noise is removed. Pre-trained models ResNet101, VGG16, VGG19, and Inception-v3 have been utilized to analyze this type of dataset.

- VGG16 Architecture

The VGG16 model was developed in 2014 by Simonyan and Zisserman. It is used in many applications due to its very uniform architecture. The number of parameters used in VGG16 is huge, at 138 million. The usage of numerous successive convolutional layers is one of the architecture’s main features. The kernel size in VGG-16 is 3 × 3, which appears to be less than in other CNN architectures. By using deep knowledge, this little 3 × 3 kernel aids in driving patterns.

- ResNet Architecture

Kaimimg proposed the ResNet architecture with his team in 2016, and ResNet took first place in the ILSVRC 2015 competition. This architecture consists of 152 layers. Batch normalization was also adopted in this model. 

ResNet-101 extracted features from the fully connected layer (fc1000). A single image’s feature vector dimension is 1 × 1000. More convolutional layers are applied to frames in the deep ResNet model to acquire stronger feature representations, which aids in improved classification outcomes. This is the fundamental distinction between the deep ResNet model and other simple neural network models.

- VGG19

The VGG-19 is a variant of the VGG-16. The ImageNet database trained the VGG-19 convolutional neural network on over a million images. The network comprises 19 layers and can categorize images into 100 distinct object classes.

- Inception-v3

By using the Inception-v3 model, a transfer learning-based method for abnormal driver behavior recognition and classification is introduced, considerably reducing the number of training data and computation costs. 

## 5. Experimental Results and Discussion

This section contains the experimental data acquired following a thorough experiment and empirical analysis of the proposed methodology for detecting abnormal behaviors of the driver. The evaluation of each strategy is explored separately in the following sections. 

### 5.1. Performance Evaluation Measures 

This paper uses four generally used evaluation metrics [27]: Precision, Recall, F1-Score, and Accuracy. The following equation used to calculate

(4) $$\mathrm{Precision}=\frac{TN}{TN+FP}$$

(5) $$\mathrm{Recall}=\frac{TP}{TP+FN}$$

(6) $$F1\_\mathrm{measure}=\frac{2\times Precision\times Recall}{Preciosion+Recall}$$

(7) $$\mathrm{Accuracy}=\frac{TP+TN}{TP+TN+FP+FN}$$

where *TP* (True Positives) is the accurately classified positive class; *FP* (False Positive); is a negative class that was inadvertently categorized as a positive class; *TN* (True Negatives) is the accurately classified negative class; *FN* (False Negative) is a positive class that was mistakenly classified as a negative class. 

### 5.2. Results Analysis of Pre-Trained and Fine-Tuned CNN Models

Convolutional neural networks trained from scratch are generally prone to errors, and fine-tuning can quickly converge the network to an ideal state, but there is a significant difference between the target dataset and the pre-trained dataset. It is difficult to extract the visual features of the image using the pre-trained CNN model in the target dataset recognition task. They fine-tuned the CNN models based on the target dataset to make the pre-trained CNN parameters more appropriate to the target dataset.

Transfer learning in machine learning has many benefits, including effective model training and resource conservation. We sought to learn and review transfer learning concepts through this study, specifically to compare the performance of pre-trained models to a CNN model created and the ResNet101, VGG16, VGG19, Inception-v3, and CNN models. While VGG16, VGG19, and ResNet101, Inception-v3 were pre-trained on the imagenet and compiled on this dataset’s driver abnormal behaviors, the CNN model underwent extensive hyper-parameter tuning to improve accuracy with the higher number of epochs and even higher resource usage. In this study, CNN results outperform the pre-trained models.

Visualization of the Confusion matrix, Classification report, AUC, model accuracy, and model loss of the pre-trained models ResNet101, VGG16, VGG19, Inception-v3 and proposed CNN-based models are shown in the following section.

**Figure 6:**
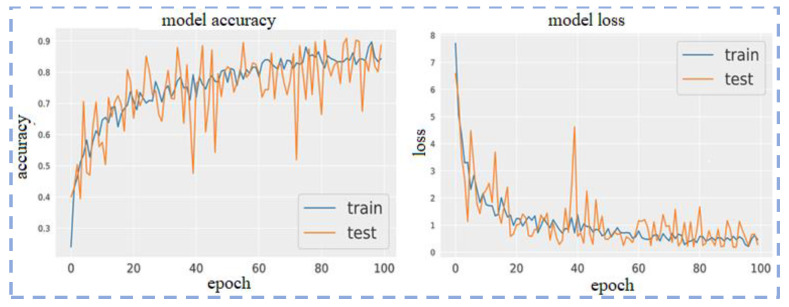
Model loss and model accuracy using ResNet101.

The loss function and optimizer used to train the model are mean squared error and Adam, respectively. Model accuracy and model loss are shown in the following figure, which shows the overfitted nature of the ResNet101 model that causes inconsistent output with poor accuracy. The model loss and model accuracy is shown in Figure 6. 

**Figure 7:**
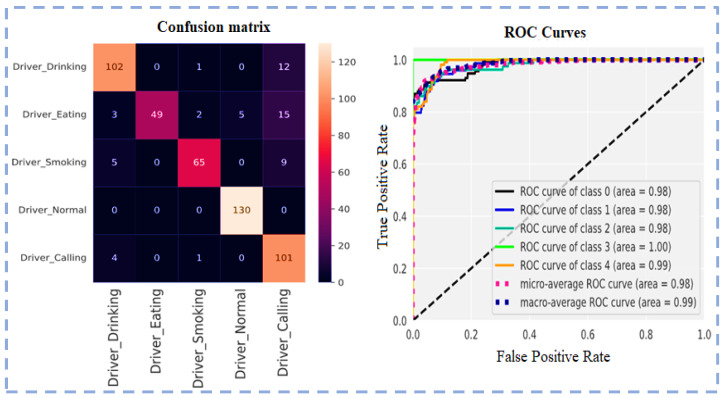
Confusion matrix and ROC Curves using ResNet101.

The confusion matrix and ROC curve using ResNet101 for five classes are shown in Figure 7.

**Figure 8:**
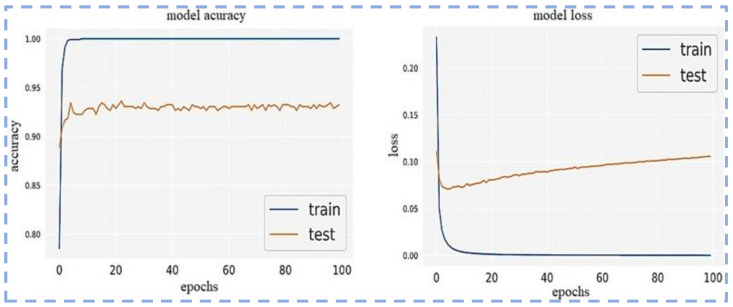
Model loss and Model accuracy using VGG16.

Next, the VGG16 pre-trained model is utilized on the given datasets, which shows the overfitted nature of the VGG16 model that causes inconsistent output with poor accuracy. However, the results demonstrate that the VGG16 loss function is more finely tuned than ResNet101 on this newly created dataset. Additionally, overfitting is somewhat reduced. The VGG16 performs comparably well to the ResNet101 pre-trained model. The model loss and model accuracy are shown in Figure 8.

**Figure 9:**
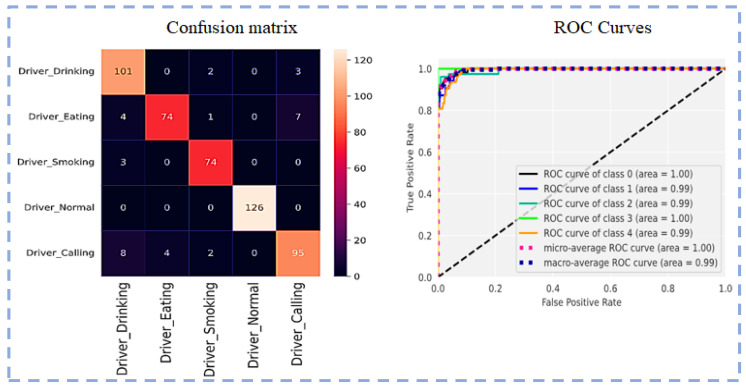
Confusion matrix and ROC Curves using VGG16.

Figure 9 shows the Confusion matrix and ROC Curves using VGG16.

**Figure 10:**
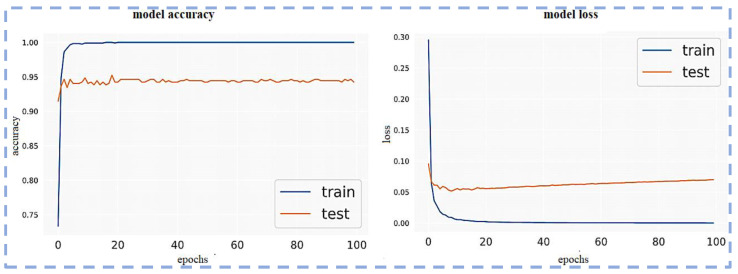
Model loss and model accuracy using VGG19.

Figure 10 illustrates the model accuracy and model loss by using VGG19. The diagram demonstrates that the VGG19 loss function is more finely tuned than VGG16 on this newly created dataset. Additionally, overfitting is somewhat reduced; this model VGG19 performs comparably well to the VGG16 pre-trained model.

**Figure 11:**
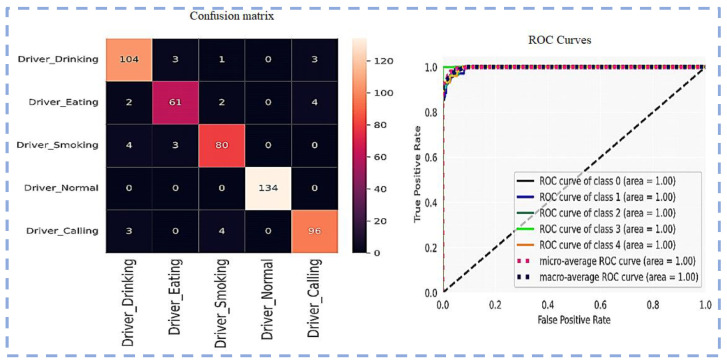
Confusion matrix and ROC Curves using VGG19.

Figure 11 displays the VGG19 ROC Curves and Confusion matrix.

**Figure 12:**
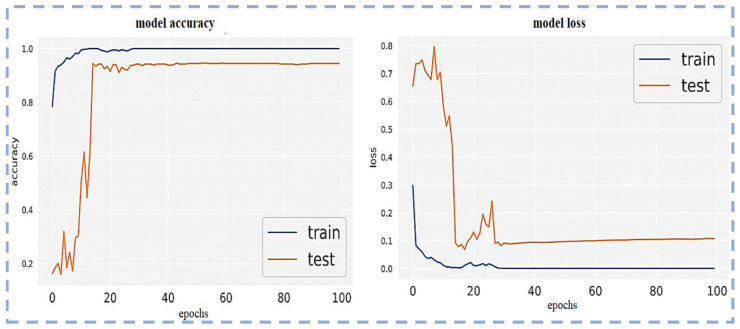
Model loss and model accuracy using Inception-v3.

Figure 12 uses Inception-v3 to demonstrate the model accuracy and model loss. The diagram shows that the pre-trained model Inception-v3 on the dataset is less precisely tuned than the VGG19 loss function. Additionally, overfitting is essentially not reduced.

**Figure 13:**
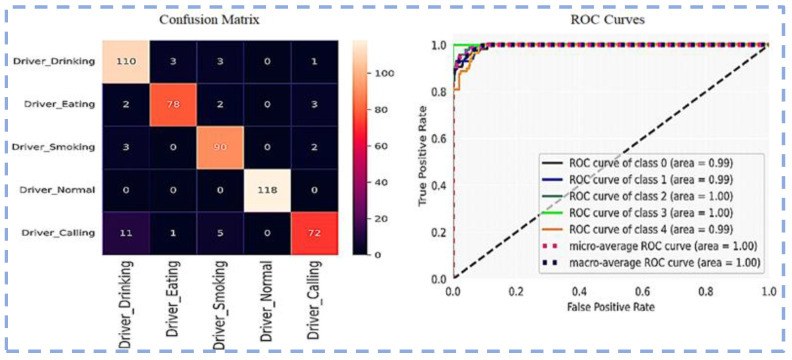
Confusion matrix and ROC Curves using Inception-v3.

Figure 13 shows the Confusion matrix and ROC Curves using Inception-v3.

**Figure 14:**
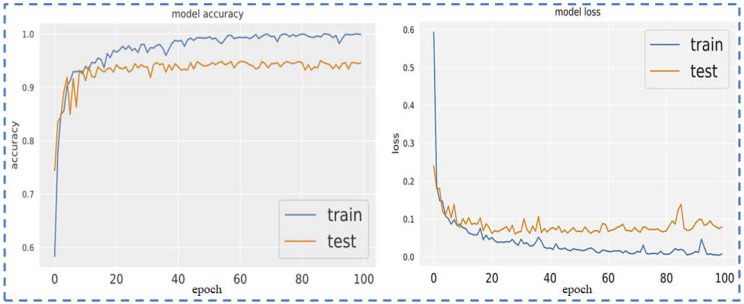
Model loss and model accuracy using the proposed CNN-based model.

**Figure 15:**
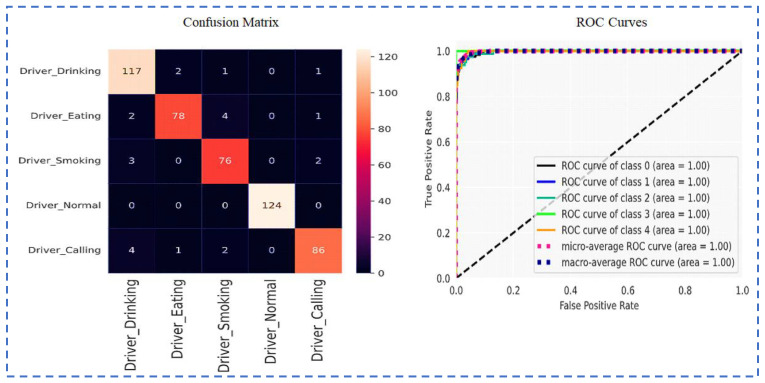
Confusion matrix and ROC Curves using the proposed CNN-based model.

Figure 14 and Figure 15 show the Confusion matrix and ROC Curves using utilized CNN model. 

### 5.3. Comparison Value of Accuracy, Precision, Recall, and F1-Measure Using Proposed CNN based and Pre-Trained Models

The value of Accuracy, Precision, Recall and F1-Score using the 20, 40, 60, 80 and 100 epochs are shown in Table 2. 

The comparison value of Accuracy, Precision, Recall, F1-measure and support value for the five classes using the proposed CNN-based model and pre-trained models ResNet101, VGG-16, VGG-19, Inception-v3 are given below in Table 3.

**Figure 16:**
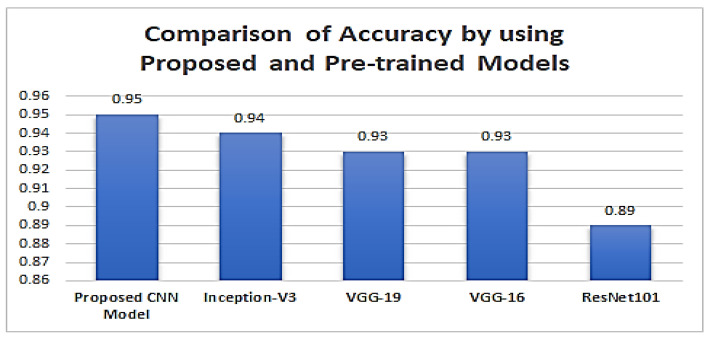
Comparison of Accuracy Values.

Figure 16 shows the accuracy values achieved by using different pre-trained models and the Our proposed CNN-based model. 

**Figure 17:**
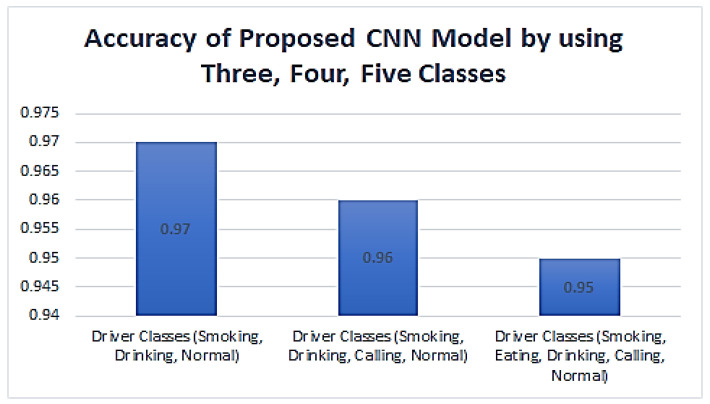
Accuracy values achieved by using three, four, and five classes.

Figure 17 shows the accuracy values achieved using the proposed CNN-based model’s three, four, and five-classes drivers. 

## 6. Conclusions

The detection of unusual driving behavior is growing more popular since it is critical in guaranteeing the safety of drivers and passengers in vehicles. Road accidents occur for various reasons, including medical problems, mental stress, weariness, and abnormal behaviors of drivers. To increase driving safety, detecting abnormal driving behaviors in real-time is necessary to increase driver awareness of their driving habits and hence reduce future road accidents. This study uses deep learning and a convolutional neural network to present an autonomous detection system for recognizing abnormal driver actions. The main advantages of this research are that the most common activities that lead to distracted drivers have been considered in this dataset creation. Only the keyframes of the dataset have been taken in this approach to reduce computational resources. All redundant frames were not considered in the processing. It helps the driver avoid on-road accidents and increases their attention on driving. Vehicle safety, as in modern age vehicles, are developing systems to avoid accidents due to human error. So, the development system must include all those activities which distract the driver and alert the driver on time. The key difficulties with the recognition model include fluctuations in illumination, pose, variations in view angle, camera position, and occluded images, which also negatively impact the approaches’ performance.

This system is trained and evaluated using a new dataset with five classifications. Driver-smoking, Driver-eating, Driver-drinking, Driver-calling, and Driver-normal are the main groups. Deep CNN and pre-trained models ResNet101, VGG-16, VGG-19, and Inception-v3 are used. The performance measures are used to compare the results. The following percentages were obtained: 89% by ResNet101, 93% by using VGG-16, 93% by using VGG-19, 94% by Inception-v3, and 95% by using the proposed CNN-based model. The obtained accuracy is decreased by 2% to 3% with the raw images (unprocessed). 

Future work will include a few additional unusual behaviors that may be connected to a driver’s health or depression levels. Depending on the visible area of the driver’s actions, as limitations. Driver activities will also be identified if they are partially displayed.

## Figures and Tables

**Table 1:** Datasets details of Unusual Activities of Drivers while driving.

Sr. No.	Name of Class	No. of Videos	No. of Frames	Key-Frames
1	Driver-smoking	6	2400	315
2	Driver-eating	7	2020	486
3	Driver-drinking	7	1400	260
4	Driver-calling	8	1800	393
5	Driver-normal	8	1500	429

**Table 2:** Accuracy, Precision (P), Recall(R) and F1-Score using the 20, 40, 60, 80 and 100 epochs.

Class Name	P	R	F1-Score	P	R	F1-Score	P	R	F1-Score	P	R	F1-Score	P	R	F1-Score
	No of Epochs = 20			No of Epochs = 40			No of Epochs = 60			No of Epochs = 80			No of Epochs = 100		
Driver-Drinking	0.89	0.90	0.90	0.87	0.96	0.91	0.99	0.89	0.94	0.94	0.92	0.93	0.93	0.97	0.95
Driver-Eating	0.73	0.86	0.79	0.88	0.90	0.89	0.88	0.93	0.90	0.92	0.87	0.90	0.96	0.92	0.94
Driver-Smoking	0.66	0.91	0.76	1.00	1.00	1.00	0.96	1.00	0.98	0.99	1.00	1.00	0.92	0.94	0.93
Driver-Normal	1.00	0.93	0.96	0.95	0.81	0.97	0.91	0.83	0.87	0.83	0.99	0.90	1.00	1.00	1.00
Driver-Calling	0.96	0.54	0.70	0.87	0.96	0.91	0.91	0.83	0.87	0.96	0.87	0.92	0.96	0.92	0.94
Accuracy	0.84			0.92			0.93			0.93			0.95		

**Table 3:** Comparison value of Accuracy, Precision, Recall, and F1-measure for different models.

Model Name	Class Name	Precision	Recall	F1-Score	Support
ResNet101	Driver-Drinking	0.89	0.89	0.89	115
	Driver-Eating	1.00	0.66	0.80	74
	Driver-Smoking	0.94	0.82	0.88	79
	Driver-Normal	0.96	1.00	0.98	130
	Driver-Calling	0.74	0.95	0.83	106
	Accuracy 0.89				
VGG-16	Driver-Drinking	0.87	0.95	0.91	106
	Driver-Eating	0.95	0.86	0.90	86
	Driver-Smoking	0.94	0.96	0.95	77
	Driver-Normal	1.00	1.00	1.00	126
	Driver-Calling	0.90	0.87	0.89	109
	Accuracy 0.93				
VGG-19	Driver-Drinking	0.87	0.94	0.91	117
	Driver-Eating	0.95	0.92	0.93	85
	Driver-Smoking	0.90	0.95	0.92	95
	Driver-Normal	1.00	1.00	1.00	118
	Driver-Calling	0.92	0.81	0.86	89
	Accuracy 0.93				
Inception-v3	Driver-Drinking	0.88	0.95	0.91	107
	Driver-Eating	0.93	0.87	0.90	85
	Driver-Smoking	0.99	0.90	0.94	90
	Driver-Normal	1.00	1.00	1.00	130
	Driver-Calling	0.90	0.93	0.91	92
	Accuracy 0.94				
Ours	Driver-Drinking	0.93	0.97	0.95	121
	Driver-Eating	0.96	0.92	0.94	85
	Driver-Smoking	0.92	0.94	0.93	81
	Driver-Normal	1.00	1.00	1.00	124
	Driver-Calling	0.96	0.92	0.94	93
	Accuracy 0.95				

## Data Availability

The data presented in this study are available upon request from the corresponding author.
